# Severe exacerbations in moderate-to-severe asthmatics are associated with increased pro-inflammatory and type 1 mediators in sputum and serum

**DOI:** 10.1186/s12890-019-0906-7

**Published:** 2019-08-08

**Authors:** Michael A. Ghebre, Pee Hwee Pang, Dhananjay Desai, Beverley Hargadon, Chris Newby, Joanne Woods, Laura Rapley, Suzanne E. Cohen, Athula Herath, Erol A. Gaillard, Richard D. May, Chris E. Brightling

**Affiliations:** 1 Institute for Lung Health NIHR Leicester Biomedical Research Centre Department of Respiratory Sciences, University of Leicester and University Hospitals of Leicester NHS Trust, Leicester, LE3 9QP UK; 2 Department of Respiratory and Critical Care Medicine, Tan Tock Seng Hospital, Singapore, Singapore; 3 MedImmune Ltd, Milstein Building, Granta Park, Cambridge, CB21 6GH UK; 4 Present address: Camallergy, Cambridge Biomedical Campus, Cambridge, UK

**Keywords:** Asthma, Cytokines, Sputum, Biomarkers

## Abstract

**Background:**

Asthma is a heterogeneous disease and understanding this heterogeneity will enable the realisation of precision medicine. We sought to compare the sputum and serum inflammatory profiles in moderate-to-severe asthma during stable disease and exacerbation events.

**Methods:**

We recruited 102 adults and 34 children with asthma. The adults were assessed at baseline, 3, 6, and 12-month follow-up visits. Thirty-seven subjects were assessed at onset of severe exacerbation. Forty sputum mediators and 43 serum mediators were measured. Receiver-operator characteristic (ROC) curves were constructed to identify mediators that distinguish between stable disease and exacerbation events. The strongest discriminating sputum mediators in the adults were validated in the children.

**Results:**

The mediators that were significantly increased at exacerbations versus stable disease and by ≥1.5-fold were sputum IL-1β, IL-6, IL-6R, IL-18, CXCL9, CXCL10, CCL5, TNFα, TNF-R1, TNF-R2, and CHTR and serum CXCL11. No mediators decreased ≥1.5-fold at exacerbation. The strongest discriminators of an exacerbation in adults (ROC area under the curve [AUC]) were sputum TNF-R2 0.69 (95% CI: 0.60 to 0.78) and IL-6R 0.68 (95% CI: 0.58 to 0.78). Sputum TNF-R2 and IL-6R were also discriminatory in children (ROC AUC 0.85 [95% CI: 0.71 to 0.99] and 0.80 [0.64 to 0.96] respectively).

**Conclusions:**

Severe asthma exacerbations are associated with increased pro-inflammatory and Type 1 (T1) immune mediators. In adults, sputum TNF-R2 and IL-6R were the strongest discriminators of an exacerbation, which were verified in children.

**Electronic supplementary material:**

The online version of this article (10.1186/s12890-019-0906-7) contains supplementary material, which is available to authorized users.

## Background

Asthma affects over 300 million people worldwide. Severe asthma represents about 10% of all asthmatics [1]. This group has the greatest unmet need with persistent symptoms, airflow obstruction, and chronic inflammation punctuated by episodes or worsening of symptoms known as exacerbations. There is an increasing recognition that asthma, in particular severe asthma, is a heterogeneous condition with variability in clinical expression of disease, disordered airway physiology, inflammation and frequency of exacerbations [1–4].

Comparisons of cytokine profiles in eosinophilic versus non-eosinophilic and Type 2 (T2)^high^ versus T2^low^ asthma and between asthma, chronic obstructive pulmonary disease (COPD) and asthma/COPD overlap have provided insights into potential underlying mechanisms and responses to therapy [5–8]. Indeed this approach has revealed that asthmatics with eosinophilic, T2^high^ disease respond more favourably to corticosteroids [5] and has uncovered non-eosinophilic, T2^low^ asthmatics in which other mechanisms such as exposure to pollutants or persistent infection might play a role.

Approaches to stratify cytokine profiles in stable disease have been applied widely, but to date there are only a few studies that have explored sputum inflammatory mediators at exacerbations in adults [9–13] and children [14, 15]. These studies have included small numbers of subjects, limited panels or single sputum mediators and focused mostly upon mild-to-moderate disease, which might explain the variable reports of either increased eosinophils or neutrophils, together with increased T1 or T2 cytokines. Thus surprisingly, there is a paucity of data profiling a broad array of sputum inflammatory mediators in stable disease versus severe exacerbations.

We hypothesised that the sputum and serum inflammatory mediator profiles change between stable disease and exacerbation events. To test this hypothesis, we undertook a 1-year prospective study of moderate-to-severe adult asthmatics, assessed at stable visits and at the onset of severe exacerbations. The findings were then validated in children admitted to hospital with acute asthma exacerbations.

## Methods

### Subjects

In this study 102 adults with moderate-to-severe asthma and 34 children with mild-to-severe asthma, according to the Global Initiative for Asthma (GINA) treatment step [16], were recruited from a single centre Glenfield Hospital, University Hospitals of Leicester NHS Trust, Leicester, United Kingdom. The adults were assessed at baseline, 3, 6, and 12-month follow-up visits and at the onset of a severe exacerbation defined as requiring high dose systemic corticosteroids (≥ 30 mg a day) for 3 or more days. Some subjects had participated in an earlier cross-sectional study [8]. The mediators that best discriminated between stable state and exacerbations in the adults were validated in children with doctor-diagnosed asthma admitted to hospital (Royal Infirmary, University Hospitals of Leicester NHS Trust, Leicester, United Kingdom) with acute-severe exacerbations or during stable visits. The studies were approved by the local Leicestershire, Northamptonshire and Rutland ethics committee (08/H0406/189). Written informed consent was obtained from each subject or the subject’s legal guardian.

### Clinical assessments

Demographic, clinical and lung function data were recorded including Asthma Control Questionnaire-6 (ACQ6) score, Asthma Quality of Life Questionnaire (AQLQ) score, symptom scores using the visual analogue scale (VAS), pre- and post-bronchodilator forced expiratory volume in the first second (FEV_1_), forced vital capacity (FVC), fraction of exhaled nitric oxide (FeNO), sputum total and differential cell counts. For the paediatric group only demographics, clinical history and sputum collection was undertaken. Spontaneous sputum was collected at exacerbations and sputum was either spontaneous or induced at stable state dependent on whether subjects produced adequate sputum spontaneously.

### Sputum and blood mediator measurements

Forty mediators in sputum supernatants, and 43 mediators in serum were measured using the Meso Scale Discovery Platform (MSD® Gaithersburg, MD, USA) and enzyme-linked immunosorbent assay (ELISA) as described previously [8]. MSD is an immunoassay that combines electrochemiluminescence in a multi-array platform. The lower limits of detection and quantification were reported in the Additional file 1: Table S1. Samples in which mediator concentrations were below half of the limit of quantification (BLQ) were assigned half the limit of quantification. Mediators that were BLQ in more than 60% of samples were assigned BLQ as a group. The groups that were assigned BLQ were not included in further analyses. These were IL-4, IL-9, IL-10, IL-12p70, IL-13, IL-17, IL-23, IL-33, GMCSF, IFN**γ,** TSLP, NGF and CEACAM-5. Serum IL-6R, CCL3, CCL5 and CCL13 were above the limit of quantification (ALQ) in all the samples and were therefore excluded from further analysis. Seventeen of the serum mediators included in the analysis had < 5% of the samples outside the measurable range and 5 (IL-1β, CCL2, TNFα, VEGF, periostin) had 5–20% of the samples outside of the measurable range. Sixteen of the sputum mediators included in the analysis had < 5% of the samples outside the measurable range and 10 (IL2, IL5, IL15, IL18, CCL3, CCL4, CCL5, CCL11, CXCL11 and TNFα) had 5–30% of the samples outside of the measurable range.

### Statistical analysis

All analyses were done with STATA/IC version 14.0 for Windows (Stata Corp, College Station, TX, USA) and Prism version 7.00 for Mac OS X (GraphPad Software, La Jolla California, USA). The parametric and log transformed data were presented as mean with Standard Error of the Mean (SEM) and geometric mean with 95% confidence interval (CI) respectively. The changes of characteristics within and between subjects were examined using paired and unpaired t-tests. Receiver operating characteristic (ROC) curves were used to identify the inflammatory mediators that significantly discriminated between stable versus exacerbation state for adults. The mediators that best discriminated between stable state and exacerbations in the adults were validated in the stable state versus exacerbation paediatric groups. No corrections were made for multiple comparisons. *P* < 0.05 was the threshold for significance.

## Results

**Fig. 1:**
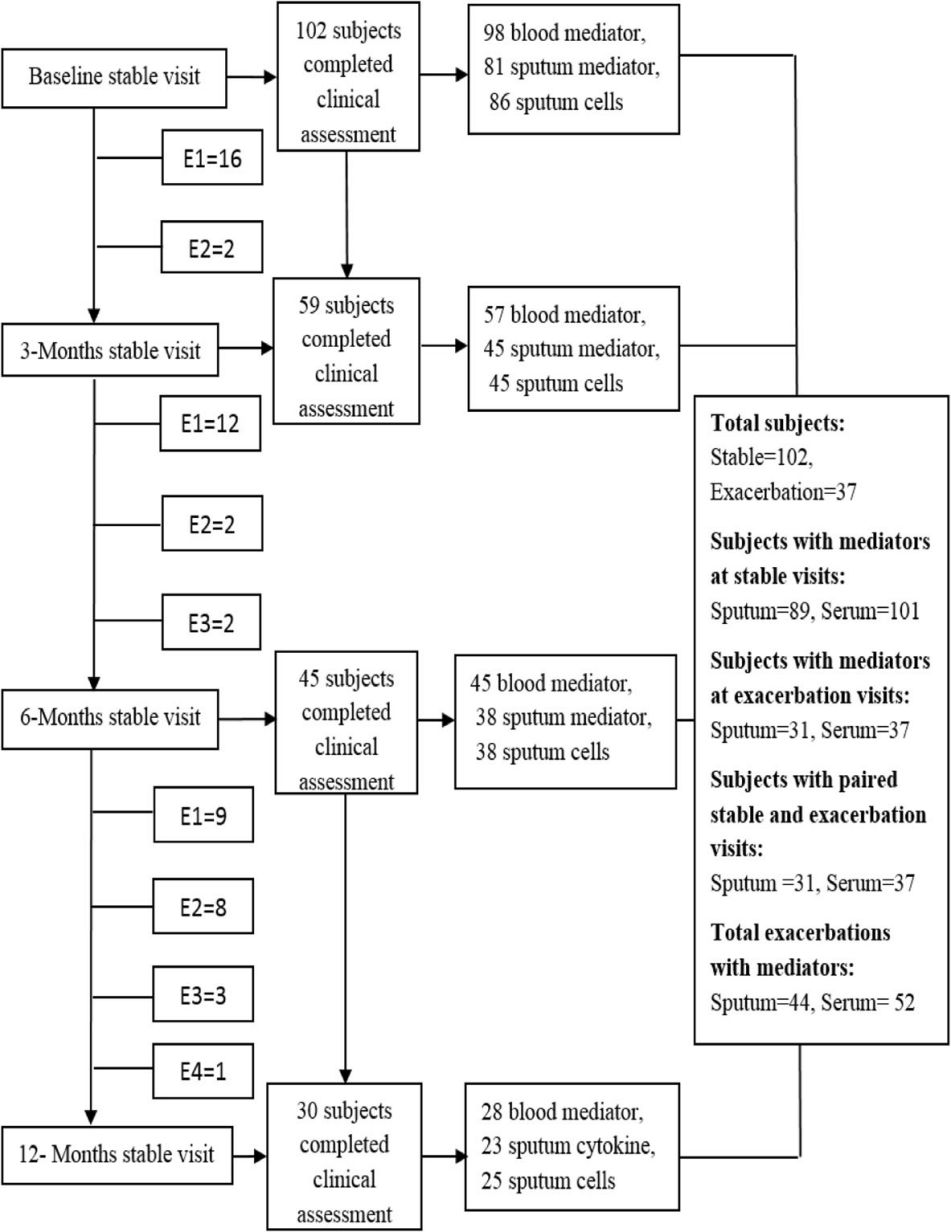
Study flow chart illustrating the number of subjects at each stable visit and intervening exacerbation (E) with corresponding number of available samples

The study flow chart is shown in Fig. 1. A total of 102 adult subjects were recruited, of which 37 subjects were assessed at one or more exacerbation visits. The clinical characteristics at baseline of all the subjects and those that were or were not assessed at an exacerbation are described in Table 1. Subjects that had an exacerbation visit had a higher body mass index (BMI) and a higher proportion of severe asthma (Step 5 according to the GINA guidelines). However, there were no significant differences in the ACQ6 score, AQLQ score, daily inhaled and oral corticosteroid dose between the two groups. The proportion of current or ex-smokers was lower in those subjects assessed at exacerbation visits. At exacerbation, there was a significant decrease in FEV_1_ and an increase in VAS cough, dyspnoea and wheeze. No significant difference was observed in blood and sputum eosinophil and neutrophil cell counts, although sputum total cell count increased significantly at exacerbation (Table 1). The clinical characteristics of the 35 children recruited with an acute-severe exacerbation (*n* = 18) or during a stable visit (*n* = 17) are as shown (Additional file 1: Table S2).

**Table 1:** Clinical characteristics at baseline and exacerbation for all subjects and those assessed or not assessed at exacerbation

Variable	Baseline All (*n* = 102)	Baseline for subjects not assessed at exacerbation (*n* = 65)	Baseline for subjects assessed at exacerbation (*n* = 37)	*P*-value∥	Change between Stable and Exacerbation (*n* = 37)	*P*-value¶
Male (n [%])	49 (48)	33 (51)	16 (43)	0.47		
Age (year)	53 (1)	52 (2)	55 (2)	0.21		
BMI (kg/m^2^)	30 (1)	29 (1)	32 (1)	0.034		
Current or Ex-smokers (n [%])	39 (38)	31 (48)	8 (22)	0.009		
Pack years^b,c^	10 (2 to 14)	10 (2 to 14)	9 (2 to 16)	0.73		
Exacerbations (OCS courses in last year) ^b^	3 (2 to 4)	2.5 (2 to 3)	3 (2 to 5)	0.21		
GINA Classification III/IV/V (n)	5/45/52	3/37/25	2/8/27	0.002		
Daily ICS dose (BDP eq μg/day) ^b^	1600 (1000 to 2000)	1600 (1000 to 2000)	1600 (800 to 2000)	0.4		
Daily Prednisolone dose (mg) ^b,d^	10 (7.5 to 15)	10 (5 to 10)	10 (7.5 to 15)	0.2		
Asthma Quality of life Questionnaire (AQLQ)	4.82 (0.14)	4.77 (0.16)	4.91 (0.25)	0.62		
Asthma Control Questionnaire-6 (ACQ-6)	1.95 (0.13)	1.97 (0.17)	1.92 (0.2)	0.87		
Atopy (n [%])	48 (47)	28 (43)	20 (54)	0.29		
IgE^a^	143 (106 to 194)	143 (96 to 212)	144 (88 to 234)	0.98		
Pre FEV_1_/FVC ratio (%)	68.4 (1.4)	68.7 (1.9)	68.0 (1.9)	0.81		
Pre FEV_1_ (L)	2.14 (0.07)	2.23 (0.09)	2.00 (0.12)	0.13	−0.15 (0.05)	0.007
Post FEV_1_ (L)	2.31 (0.08)	2.39 (0.09)	2.18 (0.14)	0.19	−0.21 (0.07)	0.005
Pre FEV_1_ (%)	74 (2)	76 (2)	72 (4)	0.38	−5.16 (2.24)	0.03
Post FEV_1_ (%)	80 (2)	82 (3)	76 (4)	0.24	−5.99 (1.87)	0.004
VAS cough (mm)	33 (3)	35 (3)	29 (4)	0.26	34 (6)	< 0.0001
VAS dyspnoea (mm)	35 (3)	36 (3)	34 (5)	0.79	31 (5)	< 0.0001
VAS wheeze (mm)	26 (3)	27 (3)	254 (5)	0.76	28 (5)	< 0.0001
TCC (10^6^/g sputum)^a^	1.52 (1.20 to 1.93)	1.56 (1.15 to 2.12)	1.46 (0.98 to 2.16)	0.79	2.3-fold (1.1 to 4.9)	0.033
Sputum eosinophil cell count (%)^a^	1.9 (1.3 to 2.9)	2.5 (1.5 to 4.1)	1.2 (0.6 to 2.4)	0.093	1.0-fold (0.4 to 2.1)	0.91
Sputum neutrophil cell count (%)	62 (3)	60 (3)	65 (4)	0.32	−3 (6)	0.67
Blood eosinophil cell count (× 10^9^ cells/L)^a^	0.24 (0.20 to 0.29)	0.26 (0.21 to 0.31)	0.21 (0.14 to 0.32)	0.31	0.70-fold (0.41 to 1.20)	0.19
Blood neutrophil cell count (×10^9^ cells/L)	5.7 (0.2)	5.3 (0.3)	6.2 (0.4)	0.076	0.38 (0.36)	0.3
Fraction of exhaled Nitric Oxide (FeNO) (ppb)^a^	23 (20 to 27)	21 (18 to 25)	27 (20 to 35)	0.13	1.3-fold (0.9 to 1.8)	0.1

Data presented as mean (standard error of mean [SEM]) unless stated; ^a^geometric mean (95% CI); ^b^median (first and 3rd quartiles); ^c^pack year history for current and ex-smokers; ^d^dose of prednisolone in those receiving prednisolone; ∥*P*-value for unpaired comparison within baseline (stable) between these who assessed and not assessed at exacerbation; ¶*P*-value for paired comparison between stable (baseline) and exacerbation visits. *Abbreviations*: *OCS* Oral corticosteroid, *ICS* Inhaled corticosteroid, *BDP eq* Beclomethasone dipropionate equivalent, *VAS* Visual analogue scale, *BMI* Body Mass Index, *FEV1* Forced Expiratory Volume in the First Second, *FVC* Forced Vital Capacity

Forty-four sputum and 52 serum samples were obtained from all the exacerbation visits, and 187 sputum and 228 serum samples were also obtained from all the stable visits. In the comparison between all stable and all exacerbation visits (Table 2), sputum IL-1β, IL-2, IL-6, IL-6R, IL-18, CXCL9, CXCL10, CCL5, TNFα, TNF-R1, TNF-R2, CHTR, and serum IL-18, CXCL10 and CXCL11 were significantly increased at exacerbations. Many T2 cytokines (IL-4, IL-9, IL-13, IL-33, TSLP) were below the limit of quantitation both at stable and exacerbation. Serum periostin was significantly decreased at exacerbation compared to stable visits. The difference between stable and exacerbation visits was less than 1.5 fold for sputum IL-2, serum IL-18, CXCL10 and periostin.

**Table 2:** Geometric mean (95%CI) sputum and serum mediator concentration (pg/ml) for all stable and all exacerbation vissits

	Sputum			Serum		
	Stable (*n* = 187)	Exacerbation (*n* = 44)	*P*-value	Stable (*n* = 228)	Exacerbation (*n* = 52)	*P*-value
IL-1α	48 (40 to 57)	50 (29 to 87)	0.85	BLQ	BLQ	
IL-1β	59 (47 to 74)	130 (66 to 253)	**0.01↑**	3.6 (3.1 to 4.1)	2.8 (2.1 to 3.8)	0.13
IL-2	0.7 (0.6 to 0.8)	1.1 (0.6 to 1.9)	**0.02↑**	BLQ	BLQ	
IL-5	2.5 (1.9 to 3.2)	2.5 (1.5 to 4.1)	0.99	4.3 (3.8 to 4.9)	4.2 (3.2 to 5.5)	0.89
IL-6	31 (25 to 40)	69 (39 to 123)	**0.01↑**	BLQ	BLQ	
IL-6R	195 (169 to 226)	402 (280 to 577)	**< 0.0001↑**	ALQ	ALQ	
IL-8	2825 (2295 to 3479)	4507 (3045 to 6671)	0.05	14 (13 to 15)	13 (11 to 16)	0.62
IL-15	1.6 (1.3 to 1.9)	2.3 (1.5 to 3.5)	0.05	BLQ	BLQ	
IL-18	25 (22 to 29)	48 (30 to 76)	**0.001↑**	323 (304 to 344)	379 (340 to 422)	**0.02↑**
CXCL9	526 (406 to 681)	1432 (801 to 2558)	**0.001↑**	68 (62 to 75)	82 (66 to 102)	**0.081**
CXCL10	632 (506 to 788)	1220 (723 to 2056)	**0.01↑**	120 (112 to 129)	159 (130 to 195)	**0.002↑**
CXCL11	39 (30 to 51)	62 (27 to 140)	0.17	135 (125 to 146)	202 (161 to 252)	**< 0.0001↑**
CCL2	262 (225 to 305)	375 (258 to 544)	0.05	621 (590 to 653)	567 (509 to 633)	0.13
CCL3	27.7 (23.0 to 33.3)	27.3 (16.8 to 44.2)	0.95	ALQ	ALQ	
CCL4	369 (288 to 473)	574 (328 to 1004)	0.13	192 (179 to 206)	179 (153 to 211)	0.42
CCL5	6.6 (5.7 to 7.8)	16.3 (9.9 to 26.7)	**< 0.0001↑**	ALQ	ALQ	
CCL11	48 (41 to 57)	41 (29 to 59)	0.44	792 (739 to 848)	785 (668 to 923)	0.92
CCL13	18 (15 to 20)	14 (11 to 19)	0.19	ALQ	ALQ	
CCL17	30 (25 to 36)	21 (15 to 31)	0.12	587 (513 to 672)	582 (455 to 746)	0.96
CCL26	8.6 (6.9 to 11.0)	8.7 (5.5 to 14)	0.95	12.4 (10.5 to 14.8)	15.4 (10.0 to 23.7)	0.31
TNFα	2.5 (2.0 to 3.2)	9.6 (4.3 to 21.3)	**< 0.0001↑**	5.1 (4.4 to 5.8)	5.3 (4.0 to 6.9)	0.78
TNF-R1	424 (360 to 500)	817 (531 to 1255)	**0.001↑**	4044 (3850 to 4247)	4004 (3677 to 4360)	0.86
TNF-R2	200 (164 to 243)	579 (355 to 943)	**< 0.0001↑**	5407 (5122 to 5708)	5498 (4886 to 6187)	0.79
VEGF	1282 (1161 to 1417)	1289 (1006 to 1651)	0.97	767 (690 to 853)	730 (569 to 929)	0.67
EGF	424 (371 to 485)	418 (332 to 528)	0.92	487 (439 to 541)	387 (276 to 543)	0.088
SCF	BLQ	BLQ		90 (84 to 97)	92 (80 to 105)	0.83
ST2	BLQ	BLQ		85.7 (77.4 to 94.9)	105 (83 to 134)	0.086
CHTR	71,358 (54,991 to 92,597)	191,549 (102,748 to 357,100)	**0.001↑**	132,039 (114,285 to 152,550)	118,860 (90,839 to 155,523)	0.54
Periostin	Not done	Not done		5.7 (5.3 to 6.2)	4.7 (3.9 to 5.7)	**0.044↓**
DDP4	Not done	Not done		449.5 (433.0 to 466.6)	418.3 (384.0 to 455.7)	0.11

*BLQ* Below limit of quantification, *ALQ* Above limit of quantification, unit of the mediators is pg/ml, ↑ increase in mediator concentration, ↓ decrease in mediator concentration

All entries in bold are significant

**Fig. 2:**
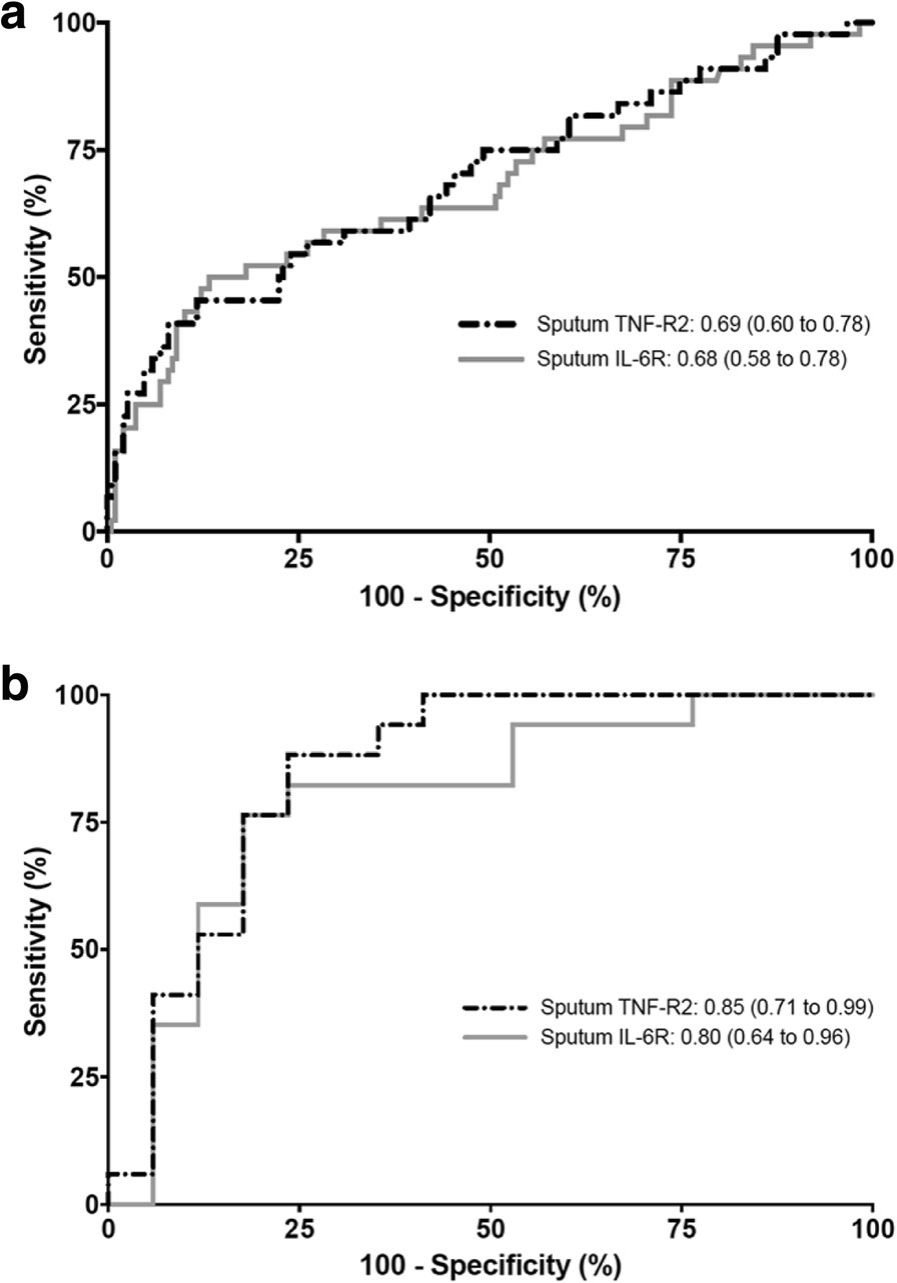
Sputum IL-6R and TNF-R2 ROC curves in adults (**a**) and children (**b**)

The ROC AUC for sputum and serum mediators between all stable and all exacerbation visits are shown in Table 3. The strongest discriminators of an exacerbation in adults were sputum TNF-R2 and IL-6R, with ROC AUC of 0.69 (95% CI: 0.60 to 0.78) and 0.68 (95% CI: 0.58 to 0.78), respectively (Fig. 2a). The results were similar in the paediatric group. The ROC AUC of sputum TNF-R2 and IL-6R in children were 0.85 (95% CI: 0.71 to 0.99) and 0.80 (95% CI: 0.64 to 0.96), respectively (Fig. 2b). In the serum, the best discriminator of an exacerbation was CXCL11, with ROC AUC of 0.66 (95% CI: 0.57 to 0.76) in adults.

**Table 3:** ROC AUC (95% CI) for sputum and serum mediators between all stable and all exacerbation visits

	Sputum	Serum
	(Stable = 187; Exacerbation = 44)	(Stable = 228; Exacerbation = 52)
IL-1α	0.50 (0.39 to 0.62)	BLQ
IL-1β	0.59 (0.49 to 0.70)	0.43 (0.35 to 0.51)
IL-2	0.57 (0.47 to 0.68)	BLQ
IL-5	0.50 (0.41 to 0.60)	0.49 (0.49 to 0.50)
IL-6	**0.61 (0.52 to 0.71)**	BLQ
IL-6R	**0.68 (0.58 to 0.78)**	ALQ
IL-8	0.59 (0.50 to 0.68)	0.50 (0.40 to 0.59)
IL-15	0.59 (0.48 to 0.69)	BLQ
IL-18	**0.62 (0.52 to 0.72)**	**0.60 (0.52 to 0.69)**
CXCL9	**0.65 (0.55 to 0.74)**	**0.60 (0.51 to 0.69)**
CXCL10	0.60 (0.50 to 0.70)	**0.61 (0.52 to 0.70)**
CXCL11	0.53 (0.43 to 0.64)	**0.66 (0.57 to 0.76)**
CCL2	0.57 (0.47 to 0.67)	0.44 (0.35 to 0.54)
CCL3	0.49 (0.38 to 0.59)	BLQ
CCL4	0.57 (0.47 to 0.67)	0.48 (0.39 to 0.57)
CCL5	**0.67 (0.56 to 0.77)**	ALQ
CCL11	0.45 (0.36 to 0.55)	0.50 (0.41 to 0.60)
CCL13	0.44 (0.36 to 0.52)	ALQ
CCL17	0.42 (0.33 to 0.51)	0.52 (0.43 to 0.60)
CCL26	0.50 (0.41 to 0.59)	0.52 (0.42 to 0.62)
TNFα	**0.64 (0.54 to 0.74)**	0.52 (0.43 to 0.61)
TNF-R1	**0.63 (0.53 to 0.73)**	0.50 (0.41 to 0.59)
TNF-R2	**0.69 (0.60 to 0.78)**	BLQ
VEGF	0.51 (0.41 to 0.62)	0.49 (0.40 to 0.58)
EGF	0.50 (0.40 to 0.59)	0.46 (0.36 to 0.55)
SCF	BLQ	0.52 (0.44 to 0.60)
ST2	BLQ	0.56 (0.47 to 0.66)
CHTR	**0.64 (0.53 to 0.74)**	0.46 (0.36 to 0.55)
Periostin	Not done	0.52 (0.46 to 0.58)
DDP4	Not done	0.41 (0.32 to 0.50)

*BLQ* Below limit of quantification, *ALQ* Above limit of quantification, unit of the mediators is pg/ml

All entries in bold are significant

Unpaired and paired comparisons of sputum and serum mediator concentrations for all first stable and first exacerbation visits are shown in Additional file 1: Tables S3 and S4, respectively. The ROC AUC for sputum and serum mediators between all first stable and first exacerbations are presented in the Additional file 1: Table S5.

## Discussion

In this study, we report the sputum and serum mediator profiles in moderate-to-severe asthmatics at stable and exacerbation visits. The results showed an increase in T1 and pro-inflammatory mediators at severe asthma exacerbations. Interestingly, there was no discernible increase in T2 mediators and, in contrast, some T2 mediators such as periostin decreased at exacerbation. From our findings, the strongest discriminators of an exacerbation were TNF-R2 and IL-6R in sputum and CXCL11 in serum. Similarly, in the paediatric group sputum TNF-R2 and IL-6R discriminated between stable and exacerbation events. Taking our findings together, up-regulated T1 and pro-inflammatory mediators rather than T2 inflammation appear to characterise severe asthma exacerbations.

TNFα is an important cytokine in the innate immune system, which is synthesized as a transmembrane precursor protein, mainly by activated macrophages and T cells. The cytoplasmic tail of this protein is then cleaved to release soluble TNFα. An aggregation of three TNFα monomers is required to form the trimeric TNFα, which then binds to ubiquitously expressed transmembrane receptors, TNF-R1 and TNF-R2. This in turn stimulates the release of various pro-inflammatory cytokines such as IL-1β, IL-6 and IL-8. TNFα has been implicated in asthma, with up-regulation of the TNFα axis noted in patients with severe refractory asthma [17–19]. However, in spite of early efficacy late phase trials of anti-TNFα therapy in asthma failed to demonstrate important benefits and were discontinued early due to more frequent infections and higher incidence of malignancies in the treatment group [20]. Soluble TNF-R1 and TNF-R2 are formed by the proteolytic cleavage of the extracellular domains of the TNFα receptors. These soluble TNFα receptors can act as antagonists and function as natural regulators of the TNFα activity, thus diminishing the pro-inflammatory effects of TNFα. In our study, both TNFα and soluble TNFα receptors are significantly up-regulated at exacerbation compared to stable visits. In this regard, IL-6R then emerges as potentially more important than TNFα and its soluble receptors.

There is emerging evidence implicating IL-6 and its receptor in the pathogenesis of asthma. Elevated levels of IL-6 had been found in serum [21], induced sputum [22], bronchoalveolar fluid [23] and lung tissues of asthmatic patients [24]. Asthmatic patients with high serum IL-6 were found to have significantly worse lung function and more frequent exacerbations [25, 26]. A large Australian genome-wide association study identified a single-nucleotide polymorphism (SNP) located in the IL-6R gene (rs4129267) to be associated with asthma risk [27]. Soluble IL-6R was also found to be elevated in serum and bronchoalveolar fluid of asthmatic patients after allergen challenge and during spontaneous exacerbation [28, 29]. There are two signalling pathways driven by IL-6. IL-6 binds to membrane-bound IL-6R (mIL-6R) found on leukocytes and hepatocytes, before associating with the gp130 glycoprotein, to trigger the intracellular signalling cascades. The trans-signalling pathway is via the soluble IL-6R (sIL-6R), which is formed by the proteolytic cleavage of the mIL-6R or the translation from alternatively spliced messenger RNA (mRNA). The IL-6/sIL-6R complex then binds to gp130 glycoprotein, which is ubiquitously expressed. Thus, in contrast to soluble TNF-R1 and 2, which antagonise the effect of TNFα, soluble IL-6R can amplify the effects of IL-6 as it permits IL-6 to stimulate cells even if they lack mIL-6R. The IL-6R coding SNP rs2228145 (Asp^358^Ala), which increases IL-6R shedding and promotes IL-6 trans-signalling is associated with lower predicted FEV_1_ [30] and is more frequent in the severe asthma clusters in the Severe Asthma Research Program (SARP) cohort. This suggests that IL-6 trans-signalling may have a key role in asthma severity. IL-6 trans-signalling has also been implicated in other diseases such as chronic inflammatory bowel disease and colon cancer [31], with a humanized monoclonal IL-6R antibody (tocilizumab) currently licensed for use in rheumatoid arthritis and systemic juvenile idiopathic arthritis [32]. IL-6 production is also linked to obesity [33]. The BMI was increased in those adult subjects that had an exacerbation versus the whole adult group whereas in contrast the BMI was lower in the exacerbation versus stable group for the children studied. In our study the increase in IL6R could not be attributed to BMI alone. Whether sputum IL-6R at exacerbations represents a target for asthma therapy or is a consequence of infection highlighting the possible need for anti-microbial therapy in some asthmatics at exacerbations is uncertain, although antibiotics have not consistently demonstrated improved outcomes for severe asthma exacerbations [34].

The interferon-γ-induced chemokines CXCL9, 10 and 11 were increased in either or both sputum and serum at exacerbations versus stable disease. These chemokines especially serum CXCL10 are increased in asthma and COPD exacerbations triggered by rhinoviral infections [6, 35, 36]. It is therefore likely that some of the exacerbation events in our study were triggered by viral infections. Both T1 and T2 responses have been reported following viral infections [9, 35, 37–39]. However, in our study there were no increases in T2 mediators. Although a number of T2 mediators (IL-4, IL-9, IL-13, IL-33, TSLP) were below half of the limit of quantitation at both stable and exacerbation states, the T2 mediator periostin decreased at exacerbation versus stable state. Taken together our findings are consistent with infection rather than perturbed T2 immunity e.g. allergic responses as an important trigger for exacerbations. Despite this, drugs targeting T2-mediated inflammation such as anti-IL-5/IL-5 receptor monoclonal antibodies are proven to reduce exacerbations in severe eosinophilic asthma [40–42]. Drugs targeting the T2 cytokines IL-4 and IL-13 also appear to play a role in reducing exacerbations in patients with severe asthma [43–45]. It remains to be determined from ongoing phase III clinical trials to what extent patient sub-groups, which specifically express elevated biomarkers indicative of IL-13 signalling, will benefit from these treatments. Our data suggest that targeting underlying T2-mediated inflammation and thereby reducing susceptibility to exacerbations may be a more likely mechanism of action than direct targeting of exacerbation triggers themselves.

There are several potential limitations in this study. We prospectively studied asthma subjects that had a good success rate of producing adequate sputum for cell differential and cytokine analysis. This could have introduced an acquisition bias towards an infective phenotype, although we did not systematically assess the aetiology of the exacerbation. Notwithstanding this limitation, our success rate as a single centre to obtain adequate sputum samples for analysis is very high. Salivary contamination can affect the interpretation of sputum mediators. However, this is minimised using standardised sputum plug selection methods. Thus, this bias is unlikely to have had a major impact on our findings. We did not record asthma age of onset which might have affected the mediator profiles. Although we undertook a relatively large study of severe asthma exacerbations compared to reported studies [9–15], the study size is modest; undertaken in a single-centre and no corrections for multiple comparisons were made. The sample size limits our ability to explore asthma subgroups using statistical techniques such as cluster analysis, which we have applied to similar datasets in COPD [6]. Importantly, we were able to examine an extensive panel of mediators in both sputum and serum samples, which allowed us to explore effects upon T1 versus T2 immunity. However, we acknowledge a need for more sensitive assays of T2 mediators especially as our groups studied did not have high levels of T2 mediators either due to the sensitivity of the assays, recruitment of a predominately T2 low group or as a consequence of corticosteroid therapy. Some of the mediators were outside the measurable range which reduces the precision of these analyses. However, the most discriminatory biomarkers namely sputum IL6R and TNFR2 were measurable in all samples. Additionally we replicated our findings in children recruited either at stale visits or at exacerbations. Although data derived from children and adults are not directly comparable the generalisability of our findings in the different age groups suggests they are not restricted to adults. Larger, multi-centre prospective studies to examine the inflammatory profiles of asthma exacerbations in adults and children are required in the future.

## Conclusions

In conclusion, we found in moderate-to-severe asthmatics that the T1 and pro-inflammatory mediators in sputum and serum were up-regulated at exacerbation without a significant T2 response. Sputum TNF-R2 and IL-6R were strongly associated with asthma exacerbations in both adults and children. Our findings suggest that the role of the IL-6/IL-6R axis in asthma exacerbations warrants further investigation.

## Additional files

Additional file 1: **Table S1.** Sputum and serum mediators lower limit of detection (LLD) and lower limit of quantification (LLQ). **Table S2.** Clinical characteristics at children assessed either at stable visits or following admission to hospital for an acute-severe exacerbation of asthma. **Table S3.** Geometric mean (95% CI) sputum mediator concentrations (pg/ml) for all first stable and first exacerbation visits. **Table S4.** Geometric mean (95% CI) serum mediator concentrations (pg/ml) for all first stable and first exacerbation visits. **Table S5.** ROC area under the curve (AUC) (95% CI) for sputum and serum mediators between first stable and first exacerbation visits. (DOCX 49 kb)

## Data Availability

The data that support the findings of this study are available from the corresponding author upon reasonable request.

## Abbreviations

ACQ6: Asthma Control Questionnaire-6
ALQ: Above the limit of quantification
AQLQ: Asthma Quality of Life Questionnaire
AUC: Area under the curve
BLQ: Below half of the limit of quantification
BMI: Body mass index
CI: Confidence interval
COPD: Chronic obstructive pulmonary disease
ELISA: Enzyme-linked immunosorbent assay
FeNO: Fraction of exhaled nitric oxide
FEV_1_: Forced expiratory volume in the first second
FVC: Forced vital capacity
GINA: Global Initiative for Asthma
mRNA: Messenger RNA
ROC: Receiver-operator characteristic
SARP: Severe Asthma Research Program
SEM: Standard Error of the Mean
SNP: Single-nucleotide polymorphism
T1: Type 1
T2: Type 2
VAS: Visual analogue scale
