# Supplementary material for: Severe exacerbations in moderate-to-severe asthmatics are associated with increased pro-inflammatory and type 1 mediators in sputum and serum
Source: BMC Pulm Med. 2019 Aug 8;19:144. doi: 10.1186/s12890-019-0906-7 (PMC6688375; doi:10.1186/s12890-019-0906-7)
Supplement: Supplementary file 1 — Table S1. Sputum and serum mediators lower limit of detection (LLD) and lower limit of quantification (LLQ). Table S2. Clinical characteristics at children assessed either at stable visits or following admission to hospital for an acute-severe exacerbation of asthma. Table S3. Geometric mean (95% CI) sputum mediator concentrations (pg/ml) for all first stable and first exacerbation visits. Table S4. Geometric mean (95% CI) serum mediator concentrations (pg/ml) for all first stable and first exacerbation visits. Table S5. ROC area under the curve (AUC) (95% CI) for sputum and serum mediators between first stable and first exacerbation visits. (DOCX 49 kb) [file 12890_2019_906_MOESM1_ESM.docx]

**Additional file**

**Table S1.** Sputum and serum mediators lower limit of detection (LLD) and lower limit of quantification (LLQ).

|  | **Sputum** | | **Serum** | |
| --- | --- | --- | --- | --- |
|  | LLQ | LLD | LLQ | LLD |
| **IL-1α** | 0.64 | 0.46 | 3.2 | 1.07 |
| **IL-1β** | 2.56 | 1.58 | 2.6 | 1.13 |
| **IL -2** | 0.64 | 0.453 | 3.2 | 2.8 |
| **IL-4** | 3.2 | 1.18 | 3.2 | 0.811 |
| **IL-5** | 0.64 | 0.476 | 3.2 | 0.677 |
| **IL-6** | 0.64 | 0.516 | 3.2 | 0.781 |
| **IL-6R** | 0.64 | 0.443 | 16 | 3.53 |
| **IL-8** | 2.56 | 0.617 | 2.6 | 0.818 |
| **IL-9** | 16 | 6.74 | 80 | 23 |
| **IL-10** | 3.2 | 2.17 | 3.2 | 1.07 |
| **IL-12p70** | 0.64 | 0.463 | 3.2 | 1.17 |
| **IL-13** | 16 | 6.75 | 80 | 33.5 |
| **IL-15** | 3.2 | 0.61 | 3.2 | 0.757 |
| **IL-17** | 16 | 4.25 | 16 | 8 |
| **IL-18** | 16 | 3.72 | 80 | 20.5 |
| **IL-23** | 400 | 180 | 2000 | 605 |
| **IL-33** | 10000 | 3265 | 10000 | 2000 |
| **CXCL9** | 0.64 | 0.247 | 3.2 | 0.783 |
| **CXCL10** | 12.8 | 3.2 | 12.8 | 6.92 |
| **CXCL11** | 3.2 | 1.24 | 16 | 3.26 |
| **CCL2** | 3.2 | 2.5 | 3.2 | 2.18 |
| **CCL3** | 16 | 13.2 | 80 | 25.5 |
| **CCL4** | 16 | 6.05 | 16 | 6.13 |
| **CCL5** | 3.2 | 1.21 | 16 | 4.56 |
| **CCL11** | 16 | 12.3 | 80 | 67.8 |
| **CCL13** | 16 | 13.4 | 16 | 6.05 |
| **CCL17** | 0.64 | 8.93 | 16 | 7.93 |
| **CCL26** | 3.2 | 0.932 | 3.2 | 1.28 |
| **TNF-α** | 0.64 | 0.31 | 3.2 | 0.953 |
| **TNF-R1** | 16 | 14.9 | 16 | 6.7 |
| **TNF-R2** | 0.64 | 0.524 | 80 | 0.519 |
| **VEGF** | 400 | 125 | 320 | 180 |
| **GMCSF** | 0.64 | 0.493 | 3.2 | 0.634 |
| **IFNγ** | 0.64 | 0.463 | 0.6 | 0.338 |
| **TSLP** | 80 | 2.37 | 400 | 295 |
| **EGF** | 6.4 | 2.36 | 6.4 | 1.18 |
| **NGF** | 6.4 | 2.35 | 6.4 | 1.35 |
| **SCF** | 6.4 | 3.22 | 6.4 | 3.26 |
| **ST2** | 32 | 25.6 | 160 | 40.7 |
| **CHTR** | 64 | 30.6 | 16000 | 3405 |
| **CEACAM-5** | Not done |  | 558 | 5 |
| **Periostin** | Not done |  | 4 | 2.46 |
| **DDP4** | Not done |  | 43.75 | 33.67 |

**Table S2.** Clinical characteristics at children assessed either at stable visits or following admission to hospital for an acute-severe exacerbation of asthma.

| **Variable** | **Stable**  **(n=17)** | **Acute-severe Exacerbation**  **(n=18)** | **P-value**¶ |
| --- | --- | --- | --- |
| **Male (n [%])** | 12 (71) | 13 (72) | 0.91 |
| **Age (year)** | 12 (0.7) | 9 (0.8) | 0.019 |
| **BMI (kg/m^2^)** | 21.4 (1.2) | 16.8 (0.7) | 0.003 |
| **GINA Treatment step (median [range])** | 4 (1 to 4) | 2 (1 to 4) | 0.001 |
| **Family history of asthma (n[%])** | 13 (100) | 7 (54) | 0.005 |
| **Hstory of eczema (n[%])** | 9 (56) | 9 (50) | 0.72 |

Mean (SEM) unless otherwise stated

**Table S3.** Geometric mean (95% CI) sputum mediator concentrations (pg/ml) for all first stable and first exacerbation visits.

|  | **All first stable (n=89)** | **First stable for subjects assessed at exacerbation (n=31)** | **Exacerbation (n=31)** | **P-value*** | **P-value**** |
| --- | --- | --- | --- | --- | --- |
| **IL-1α** | 51 (41 to 64) | 43 (30 to 68) | 63 (34 to 118) | 0.44 | 0.16 |
| **IL-1β** | 65 (49 to 87) | 70 (45 to 108) | 155 (68 to 353) | **0.011↑** | **0.026↑** |
| **IL-2** | 0.8 (0.6 to 1.0) | 0.8 (0.5 to 1.3) | 1.3 (0.6 to 2.5) | 0.11 | 0.26 |
| **IL-5** | 2.7 (1.9 to 3.9) | 4.4 (2.4 to 7.8) | 2.6 (1.4 to 5.1) | 0.94 | 0.19 |
| **IL-6** | 42 (30 to 59) | 50 (29 to 89) | 103 (55 to 192) | **0.01↑** | **0.042↑** |
| **IL-6R** | 207 (168 to 256) | 270 (183 to 397) | 436 (288 to 659) | **0.001↑** | **0.012↑** |
| **IL-8** | 3002 (2240 to 4024) | 3562 (2179 to 5822) | 5144 (3212 to 8237) | 0.061 | 0.09 |
| **IL-15** | 1.4 (1.1 to 1.9) | 1.8 (1.2 to 2.7) | 2.4 (1.5 to 3.9) | **0.041↑** | 0.34 |
| **IL-18** | 25.0 (19.8 to 31.0) | 28 (19 to 42) | 56 (32 to 100) | **0.001↑** | **0.004↑** |
| **CXCL9** | 567 (393 to 818) | 714 (340 to 1503) | 1806 (922 to 3538) | **0.002↑** | **0.014↑** |
| **CXCL10** | 707 (515 to 970) | 882 (491 to 1586) | 1362 (719 to 2583) | **0.047↑** | 0.28 |
| **CXCL11** | 53 (37 to 77) | 66 (34 to 129) | 67 (24 to 187) | 0.6 | 0.99 |
| **CCL2** | 274 (219 to 344) | 345 (212 to 562) | 445 (278 to 716) | **0.042↑** | 0.4 |
| **CCL3** | 312 (24 to 41) | 39 (23 to 64) | 37 (20 to 67) | 0.62 | 0.89 |
| **CCL4** | 364 (259 to 511) | 410 (227 to 741) | 688 (366 to 1292) | 0.066 | 0.12 |
| **CCL5** | 8.2 (6.4 to 11.0) | 9.6 (6.0 to 15.0) | 20 (11 to 36) | **0.001↑** | **0.014↑** |
| **CCL11** | 55 (43 to 69) | 60 (40 to 92) | 47 (30 to 73) | 0.5 | 0.33 |
| **CCL13** | 21 (17 to 26) | 26 (18 to 36) | 14.9 (11.0 to 20.0) | 0.1 | **0.009↓** |
| **CCL17** | 26 (20 to 34) | 30.2 (18.0 to 51.0) | 20.8 (13.0 to 34.0) | 0.37 | 0.22 |
| **CCL26** | 10.0 (7.4 to 14.0) | 11.0 (6.8 to 19.0) | 8.7 (4.7 to 16.0) | 0.63 | 0.42 |
| **TNFα** | 2.9 (2.0 to 4.0) | 3.7 (2.1 to 6.6) | 14.0 (5.5 to 36.0) | **<0.0001↑** | **0.004↑** |
| **TNF-R1** | 474 (375 to 600) | 606 (390 to 942) | 953 (564 to 1605) | **0.006↑** | **0.032↑** |
| **TNF-R2** | 220 (164 to 296) | 307 (180 to 524) | 673 (376 to 1203) | **<0.0001↑** | **0.005↑** |
| **VEGF** | 1373 (1163 to 1622) | 1514 (1123 to 2040) | 1441 (1092 to 1902) | 0.77 | 0.74 |
| **EGF** | 370 (298 to 458) | 362 (198 to 663) | 460 (344 to 616) | 0.22 | 0.69 |
| **CHTR** | 90798 (62916 to 131036) | 135247 (69719 to 262365) | 213581 (100828 to 452422) | **0.028↑** | 0.14 |

*****p-value for unpaired comparison between all first stable and first exacerbation visits; ******p-value for paired comparison between first stable and first exacerbation visits; ↑ increase in mediator concentration; ↓ decrease in mediator concentration.

**Table S4.** Geometric mean (95% CI) serum mediator concentrations (pg/ml) for all first stable and first exacerbation visits.

|  | **All first stable (n=101)** | **First stable for subjects assessed at exacerbation (n=37)** | **Exacerbation (n=37)** | **P-value*** | **P-value**** |
| --- | --- | --- | --- | --- | --- |
| **IL-1β** | 5.9 (4.8 to 7.1) | 6 (4.4 to 8.3) | 3.2 (2.3 to 4.6) | **0.002↓** | **0.001↓** |
| **IL-5** | 5.6 (4.4 to 7.1) | 6.1 (4.1 to 9.2) | 4.0 (2.8 to 5.5) | 0.092 | 0.07 |
| **IL-8** | 14 (12 to 15) | 14 (12 to 16) | 12.0 (9.5 to 15.0) | 0.31 | 0.32 |
| **IL-18** | 346 (313 to 382) | 406 (343 to 479) | 391 (342 to 447) | 0.16 | 0.62 |
| **CXCL9** | 63 (54 to 74) | 73 (58 to 91) | 78 (61 to 100) | 0.14 | 0.88 |
| **CXCL10** | 124 (111 to 138) | 137 (114 to 164) | 168 (132 to 214) | **0.01↑** | 0.11 |
| **CXCL11** | 129 (111 to 150) | 150 (115 to 196) | 217 (162 to 290) | **0.001↑** | 0.15 |
| **CCL2** | 660 (601 to 725) | 664 (588 to 749) | 567 (494 to 651) | 0.082 | **0.003↓** |
| **CCL4** | 197 (174 to 223) | 202 (172 to 238) | 181 (147 to 223) | 0.49 | 0.21 |
| **CCL11** | 910 (807 to 1026) | 987 (815 to 1195) | 831.9 (684.0 to 1011.0) | 0.43 | 0.082 |
| **CCL17** | 790 (636 to 982) | 949 (634 to 1418) | 644 (474 to 874) | 0.31 | **0.027↓** |
| **CCL26** | 19 (15 to 25) | 22 (15 to 33) | 18 (11 to 31) | 0.87 | 0.4 |
| **TNFα** | 6.7 (5.1 to 8.8) | 7.1 (4.8 to 10.3) | 5.7 (4.0 to 8.2) | 0.51 | 0.3 |
| **TNF-R1** | 4071 (3805 to 4357) | 4249 (3891 to 4640) | 3932 (3532 to 4378) | 0.59 | 0.052 |
| **TNF-R2** | 5509 (5138 to 5907) | 5737 (5123 to 6425) | 5233 (4544 to 6027) | 0.47 | 0.069 |
| **VEGF** | 926 (798 to 1076) | 880 (672 to 1154) | 767 (574 to 1024) | 0.21 | 0.36 |
| **EGF** | 600 (488 to 737) | 578 (382 to 875) | 412 (271 to 626) | 0.071 | 0.27 |
| **SCF** | 90 (79 to 102) | 92 (72 to 117) | 90 (75 to 107) | 0.99 | 0.81 |
| **ST2** | 88 (73 to 105) | 76 (57 to 102) | 117 (84 to 162) | 0.098 | **0.033↑** |
| **CHTR** | 128633 (103578 to 159749) | 130046 (82144 to 205880) | 127970 (90452 to 181050) | 0.98 | 0.77 |
| **Periostin** | 5.7 (5.0 to 6.5) | 6.1 (5.0 to 7.3) | 4.6 (3.7 to 5.8) | 0.12 | **0.004↓** |
| **DDP4** | 446 (421 to 472) | 433 (393 to 477) | 440 (401 to 484) | 0.82 | 0.68 |

*****p-value for unpaired comparison between all first stable and first exacerbation visits; ******p-value for paired comparison between first stable and first exacerbation visits;↑ increase in mediator concentration; ↓ decrease in mediator concentration.

**Table S5.** ROC area under the curve (AUC) (95% CI) for sputum and serum mediators between first stable and first exacerbation visits.

|  | **Sputum** | **Serum** |
| --- | --- | --- |
|  | **(Stable=89; Exacerbation=31)** | **(Stable=101; Exacerbation=37)** |
| **IL-1α** | 0.53 (0.39 to 0.67) | BLQ |
| **IL-1β** | 0.60 (0.47 to 0.74) | 0.33 (0.22 to 0.44) |
| **IL-2** | 0.57 (0.45 to 0.7) | BLQ |
| **IL-5** | 0.50 (0.37 to 0.62) | 0.37 (0.26 to 0.48) |
| **IL-6** | 0.64 (0.53 to 0.76) | BLQ |
| **IL-6R** | 0.69 (0.57 to 0.81) | ALQ |
| **IL-8** | 0.61 (0.49 to 0.73) | 0.47 (0.35 to 0.58) |
| **IL-15** | 0.62 (0.49 to 0.75) | BLQ |
| **IL-18** | 0.65 (0.53 to 0.78) | 0.59 (0.48 to 0.70) |
| **CXCL9** | 0.67 (0.56 to 0.78) | 0.64 (0.52 to 0.76) |
| **CXCL10** | 0.61 (0.48 to 0.73) | 0.63 (0.51 to 0.74) |
| **CXCL11** | 0.51 (0.37 to 0.64) | 0.71 (0.59 to 0.83) |
| **CCL2** | 0.60 (0.47 to 0.72) | 0.42 (0.31 to 0.53) |
| **CCL3** | 0.52 (0.39 to 0.66) | BLQ |
| **CCL4** | 0.61 (0.48 to 0.73) | 0.47 (0.36 to 0.59) |
| **CCL5** | 0.67 (0.55 to 0.80) | BLQ |
| **CCL11** | 0.46 (0.33 to 0.58) | 0.46 (0.35 to 0.57) |
| **CCL13** | 0.40 (0.30 to 0.51) | ALQ |
| **CCL17** | 0.43 (0.31 to 0.55) | 0.44 (0.34 to 0.55) |
| **CCL26** | 0.46 (0.34 to 0.58) | 0.49 (0.37 to 0.61) |
| **TNFα** | 0.68 (0.56 to 0.80) | 0.48 (0.38 to 0.59) |
| **TNF-R1** | 0.63 (0.51 to 0.76) | 0.45 (0.34 to 0.56) |
| **TNF-R2** | 0.70 (0.58 to 0.82) | 0.45 (0.33 to 0.58) |
| **VEGF** | 0.51 (0.39 to 0.64) | 0.44 (0.33 to 0.55) |
| **EGF** | 0.56 (0.43 to 0.69) | 0.37 (0.26 to 0.49) |
| **SCF** | BLQ | 0.53 (0.42 to 0.63) |
| **ST2** | BLQ | 0.58 (0.46 to 0.71) |
| **CHTR** | 0.61 (0.48 to 0.73) | 0.50 (0.37 to 0.62) |
| **Periostin** | Not done | 0.43 (0.30 to 0.54) |
| **DDP4** | Not done | 0.47 (0.36 to 0.58) |

BLQ = Below limit of quantification; ALQ = Above limit of quantification.
